# Effects of the Brain Wave Modulation Technique Administered Online on Stress, Anxiety, Global Distress, and Affect During the First Wave of the COVID-19 Pandemic: A Randomized Clinical Trial

**DOI:** 10.3389/fpsyg.2021.635877

**Published:** 2021-05-19

**Authors:** Mauro Cozzolino, Giovanna Celia, Laura Girelli, Pierpaolo Limone

**Affiliations:** ^1^Department of Humanities, Philosophy and Education, University of Salerno, Fisciano, Italy; ^2^Department of Economics, Management and Territory, University of Foggia, Foggia, Italy

**Keywords:** mind-body interventions, brain wave modulation technique, COVID-19, stress, anxiety

## Abstract

This study aims to evaluate the effects of an innovative mind-body practice named the brain wave modulation technique (BWM-T) on stress, anxiety, global distress, and affect. The technique was administered online through a web-based video conferencing platform. The intervention started on week four of the first quarantine in Italy (week commencing 30th March 2020), for a duration of 4 weeks and ended before lockdown measures were loosened. 310 people participated in the study, mean age 28.73 years old (*SD* = 9.16), 77.8% women. Of these, about half were randomly assigned to the experimental group and the other half served as controls. Participants completed online psychological tests before and after the intervention. 266 people (144 experimental, 122 controls) completed the post-intervention tests. Consistent with our hypothesis, the study’s findings indicate a reduction in the levels of stress, anxiety, global distress, and negative affect in the experimental group, compared to the control group. Moreover, the experimental group also showed higher levels of positive affect, compared to controls after the intervention. The present findings add to the current literature in suggesting that the BWM-T reduced stress not only when administered face-to face but also when administered online during the COVID-19 pandemic. Moreover, we also noted that the BWM-T has an effect on anxiety, global distress, and affect, which we had not investigated in previous studies.

## Introduction

To date, the COVID-19 pandemic has affected more than 126 million people worldwide, with nearly more than 2.77 million confirmed deaths [World Health Organization (WHO), 2021] and the numbers continue to rise. Current research indicates that the COVID-19 outbreaks have been accompanied by a mental health epidemic (Yao et al., 2020). Not surprisingly, the most common psychological symptoms during the pandemic include stress and anxiety (Araújo et al., 2020; Asmundson and Taylor, 2020; Galea et al., 2020; Qiu et al., 2020; Rajkumar, 2020; Zhou et al., 2020). Exposure to an overflow of alarming information from the media, social and affective isolation, loneliness, and reduced autonomy following lockdown have caused or aggravated psychological symptoms (Araújo et al., 2020; Zhou et al., 2020). Other less severe psychological problems such as panic behavior, hoarding and stockpiling of resources, fear of attending public events, concerns about job, income, and security have also been observed (Zhou et al., 2020). Likewise, the economic slowing caused by the business and industrial shutdown has negatively affected well-being and quality of life for millions of people worldwide (Araújo et al., 2020). Therefore, there is growing concern regarding the long-term psychological consequences of the pandemic and the potential damages of fear and panic in the general population (Rajkumar, 2020; Zhou et al., 2020).

Providing mental health treatment in the current context of social-distancing and isolation is a great challenge. Since face-to-face psychological interventions have become unsafe due to the fast transmission of the virus, the pandemic has determined an unprecedented shift to telehealth and e-health (Wind et al., 2020). Despite a body of research suggesting its effectiveness (Mohr et al., 2018; Berryhill et al., 2019), distance mental health treatments had long been overlooked by mental health professionals. COVID-19 pandemic has therefore speeded up (by at least a decade) the implementation of online mental health services and e-health tools in standard practice (Wind et al., 2020). The provision of e-mental health assistance through telephone, videoconferencing, smartphone applications, online forums, text-messaging etc., will likely help improve well-being and relieve the psychological implications of the pandemic (Zhou et al., 2020). These communication media have shown to be effective particularly with respect to depression, anxiety, and PTSD (Zhou et al., 2020).

During the outbreak, most affected countries have implemented online mental health measures (Araújo et al., 2020; Li et al., 2020; Lima et al., 2020; Waller et al., 2020; Xiang et al., 2020). Italy has made several telehealth and e-health services available to the population, including a governmental hotline for psychological support (Covid-19 – Numero verde di supporto psicologico 800.833.833, 2021) and mental health services for university students and medical institutions through online platforms. China has also implemented online psychological self-help intervention systems and several artificial intelligence (AI) programs, including one for suicide prevention (Liu et al., 2020).

Experts say that the impact of the pandemic will probably last much longer after the coronavirus disappearance (Araújo et al., 2020; Cao et al., 2020; Lima et al., 2020). As a consequence, managing this long-term impact requires effective evidence-based therapies that can be favorably administered online for those who will continue to have difficulties accessing face-to-face mental health consultations.

Mind–body interventions are based on several practices designed to facilitate the mind’s positive impact on the body (American Academy of Pediatrics, 2016). This definition includes the ancient practices for well-being such as meditation, yoga, tai chi and qigong, up to the most modern Western practices such as hypnotherapy, progressive relaxation, autogenic training, awareness, biofeedback, and guided imagery. The list also includes new practices developed over the past few decades such as eye movement desensitization reprocessing (Fernandez and Faretta, 2007), mind–body transformation therapy (Rossi et al., 2013), and brain wave modulation (Cozzolino and Celia, 2016), which we used for the purposes of this study. In addition, mind–body interventions often require a training that may be challenging to learn and are generally time-consuming to perform. Other reasons why people eventually dropout of such programs include conflicting time demands and discomfort working in groups (Dobkin et al., 2012). In this study, we chose a stress reduction method known as brain wave modulation (BWM; Hirai, 1975; Cozzolino and Celia, 2016; Cozzolino et al., 2018; Celia, 2020; Celia and Cozzolino, 2021), which originates from the interaction between Eastern doctrines and the scientific study of the mind–body dialogue (Cozzolino and Celia, 2016). The rationale of the technique traces back to the studies led in the Seventies by Tomio Hirai, one of the most influential Japanese psychiatrists of that age. His experimental studies on nocturnal sleep and brain waves (Hirai et al., 1968, 1969; Kasamatsu and Hirai, 1973; Hirai, 1975) highlighted the extraordinary beneficial effects of the Zazen practice (seated meditation practiced by priests and disciples of Zen sects of Buddhism) on the human brain. In particular, Hirai (1975) demonstrated that Zen meditation induced the slowing of EEG patterns parallel with the mental states and specific changes in consciousness. The BWM arises from the integration of Hirai’s findings with recent mind–body procedures (Schriner, 1990; Rossi et al., 2011; Cozzolino and Celia, 2016; Celia, 2020) under a neuroscientific and clinical approach. The technique involves an easy-to-implement 4-step finger movement procedure (see “Appendix” in Supplementary Material), and spontaneously helps our brain to release slower alpha waves (Desai et al., 2015). The BWM presents certain advantages over traditional mind–body interventions. First, it is very easy to learn and can be performed in minutes. Therefore, it is a sustainable and reproducible intervention that students might prefer over other methods that are more time-consuming and difficult to learn. Second, neither special premises nor specific equipment is required, so the BWM can be performed virtually everywhere. Third, the intervention can be administered individually as well as to a large number of subjects at the same time in a single session, as in this study, and it takes just one psychologist, which simplifies scheduling. Moreover, once the subjects have learned the technique, they can perform it autonomously. Previous study showed the positive effect that the BWM application can have in reducing the perceived level of stress as compared to other kind of session on stress management (Cozzolino and Celia, 2016; Celia, 2020; Cozzolino et al., 2020a,b). Recently, a line of research within neuroscience and psychosocial genomics (Rossi et al., 2006, 2008; Rossi and Rossi, 2007; Atkinson et al., 2010; Cozzolino et al., 2014, 2015, 2021b; Niles et al., 2014) has provided new insights into the pathophysiology of stress and its mind-body treatment.

Psychosocial genomics is a relatively new study of the modulation of gene expression in response to psychological, social and cultural experiences in everyday life (Rossi et al., 2010). These studies have identified the relationship between these experiences and special gene sets involved in a number of biological pathways, including stress response, inflammation and physical health. These gene sets include activity-dependent genes and rapid early genes that are able to rapidly respond to environmental stimuli (Rossi et al., 2006, 2011; Atkinson et al., 2010). This line of research has focused on the link between mental processes and gene expression. It is also studying if and how stress and relaxation can be modulated by using innovative, neuroscientific based mind-body therapies. As a consequence, a new class of mind-body practices has emerged, such as the mind-body transformations therapy (MBT-T), which includes the Creative Psychosocial Genomic Healing Experience (CPGHE; Rossi et al., 2013; Cozzolino et al., 2014; Muñoz and Larkey, 2018), and the Brain Wave Modulation Technique (BWM-T) in particular (Cozzolino and Celia, 2016; Cozzolino et al., 2020a,b, 2021a,b), which we used for the purposes of our study. As research shows, these practices can bring about positive gene expression changes in the genomic and epigenetic pathways of stress as well as induce significant stress reduction by using mind-body automatic processes that naturally occur in the mind and body during the daily and hourly basic rest-activity cycle (Lloyd and Rossi, 2008). Based on these studies, we hypothesized that the BWM-T might be particularly suited for administration in digital environment because, since it replicates these natural mind-body automatic processes, face-to-face human interaction is not so important and key as it is in traditional interventions. Moreover, the BWM-T has several other advantages. For example, it is a low-cost, sustainable, and reproducible procedure that can be performed in minutes; no special premises or specific equipment are required; it applies to both individuals and groups; it takes just one psychologist, which simplifies scheduling; and, once learned, individuals can perform it autonomously at home.

The BWM-T is a stress-reduction technique that originates from the study of Eastern practices and the study of the mind-body dialogue under a neuroscientific perspective (Cozzolino and Celia, 2016). It can be traced back to Tomio Hirai, an influential Japanese psychiatrist who researched on nocturnal sleep and brain waves (Kasamatsu and Hirai, 1966; Hirai et al., 1968, 1969; Hirai, 1975). Hirai (1975) showed that Zen meditation generated changes of consciousness and slower EEG patterns that corresponded with the disciples’ mental states. Therefore, the BWM-T has evolved from the integration of Hirai’s findings with neuroscientific based mind-body procedures (Schriner, 1990; Cozzolino and Celia, 2016; Cozzolino et al., 2020a,b).

The present study aims at evaluating the effects of an innovative mind-body practice named the brain wave modulation technique (BWM-T) on stress, anxiety, global distress, and affect in young people (Girelli et al., 2018, 2019). Following a recent study that showed the effects of a face-to-face BWM-T intervention on stress reduction (Cozzolino et al., 2020a,b), we investigated whether the technique could be effective in promoting general wellbeing, by reducing the levels of perceived stress, anxiety, global distress, and negative affect, and by improving positive affect. Furthermore, we explored whether the BWM-T could be effectively administered online through a web-based communication platform during the first wave of the COVID-19 outbreak in Italy. The intervention was carried out on week four of the first quarantine in Italy (week commencing 30th March 2020), for a duration of 4 weeks, and it ended before national lockdown measures were loosened (May 4th 2020).

The current COVID-19 emergency and the characteristics of the BWM-T led us to hypothesize that the online intervention that we carried out in this study would produce beneficial effects on the variables considered in the experimental group, compared to controls.

## Materials and Methods

### Sample

The sample was made up of non-clinical subjects and included university students, who attended courses at different educational levels. Participants were recruited with an e-mail announcement of the study sent to all students through academic platforms. 310 students had interest in taking part in the study. Exclusion criteria were age under 18 and severe mental or physical impairment. All the subjects were considered to be eligible. The mean age of all participants was 28.73 years old (*SD* = 9.16). Of the 310 analyzed participants, 241 were woman (77.7%), reflecting the typical demographics of the university and post-graduate courses our participants attended.

Participants were asked to complete an online questionnaire at two time-points, before the online intervention and after the intervention. The online questionnaire collected information on demographic data and psychological variables. Each participant was assigned a subject ID. After completing the questionnaires, subjects were randomly assigned to the experimental condition or the control condition. Randomization was accomplished by the use of an online tool (Research Randomizer, 2020). All subjects gave their informed consent for inclusion before they participated in the study.

The informed consent form made it clear to the participants that it was their right to opt out of the study at any time. Questionnaires were anonymous to ensure confidentiality and reliability of data. Of the 310 participants, 266 (85.8%) completed the questionnaire at both time-points (144 for the experimental and 122 for the control group). All procedures in this study were performed in accordance with the ethical standards of the Italian Association of Psychology (AIP) research committee and with the 1964 Helsinki declaration and its later amendments. No further approval was required.

### Measures

All the measures were digitalized and made accessible to participants from online forms via a link that was shared through academic platforms.

The Distress Thermometer (DT; Jacobsen et al., 2005) and the Perceived Stress Scale (PSS; Cohen et al., 1983) were used to evaluate perceived stress. The DT is a single-item screening tool that is well-validated to be sensitive and specific to the construct of stress (Snowden et al., 2014). The instrument was already found to be valid for measuring stress in the Italian context (Cozzolino et al., 2020a,b). Participants were asked to indicate their current level of stress on an 11-point scale ranging from 0 (zero) to 10 (ten), where 0 = no stress and 10 = maximum stress (Jacobsen et al., 2005). In order to corroborate this instrument, we also used a longer measure of stress. The PSS is a well-known stress assessment tool to understand how different situations affect our feelings and our perceived stress. It includes 10 questions about feelings and thoughts during the last month (Cohen et al., 1983). In the present study, since the testing was carried out at the beginning of April 2020, the preceding month coincided with the first wave of the pandemic in Italy. Several studies demonstrated the good psychometric properties of this scale also in the Italian context (i.e., Lee, 2012; Mondo et al., 2019). The Cronbach’s α of the scale for this study was 0.86. The State-Trait Anxiety Inventory, form Y (STAI-Y) was used to assess anxiety. It is a self-report measure for current symptoms of anxiety and a generalized propensity to be anxious (Spielberger et al., 1983). It comprises two subscales, one that evaluates the current state of anxiety (state anxiety scale), and the other that measures relatively stable aspects such as confidence, general states of calmness, and security (trait anxiety scale). The scale was proven to have good psychometric properties in the Italian context (Pedrabissi and Santinello, 1989). The Cronbach’s α for this study were 0.95 and 0.92 for state and trait anxiety scales, respectively. The YP-CORE is a self-report measure of subjective well-being, commonly experienced problems or symptoms, risk, and life/social functioning, and is was used as an indicator of global distress (Twigg et al., 2009; Twigg and McInnes, 2010). The scale demonstrated to have acceptable psychometric properties also in the Italian context (Palmieri et al., 2009; Twigg et al., 2009). The Cronbach’s α for this study was 0.77. The positive and negative affect schedule (PANAS) (Watson et al., 1988), was used to assess positive and negative affect. The positive affect scale indicates the level of enjoyable engagement as expressed by adjectives such as interested, enthusiastic, excited, active, and determined. The negative affect scale indicates a general dimension of unpleasant engagement and subjective distress that incorporates negative affects such as fear, anxiety, guilt, and shame (Terracciano et al., 2003; Alivernini et al., 2019). Participants rated the extent to which they had felt specific positive (e.g., enthusiastic) and negative (e.g., nervous) emotions over the past few weeks (1 = very slightly; 5 = extremely). For our participants, this time span encompassed the first quarantine period. Terracciano et al. (2003) gave support for the construct validity of the Italian version of the scale. The Cronbach’s α for this study were 0.87 and 0.85 for PA and NA, respectively. All the instruments were administered in Italian, the first language of participants. All the measures were already available (and validated) in Italian, so it was not necessary to translate them.

#### Design

We used a randomized controlled trial design with one between-subject factor and one within-subject factor. A researcher not directly involved in the intervention generated a blocked randomization list by the use of an online randomizer tool (Research Randomizer, 2020) and applied this list to the sample (Lahmann et al., 2017). After receiving informed consent from a participant, a randomization request was sent from the study clinical psychologist to the researcher and the results were returned to the clinical psychologist in due time. Based on these results, each participant was allocated to the control or the experimental condition. We randomized *n* = 137 participants to the control condition and *n* = 173 participants to the experimental condition. Although we intended to randomize half the participants to each condition, we decided to include a greater number of subjects in the experimental condition (25% more), due to the fact that in similar studies the drop-out rate for these subjects was about 25% (Deckro et al., 2002; Birdee et al., 2009; Cozzolino et al., 2020a,b).

### Intervention

The intervention was carried out online once a week, for a duration of 4 weeks. During the study, participants in the experimental condition received a 15 min online session of the BWM-T, whereas controls watched 15 min videos that gave suggestions on how to reduce stress. The BWM-T practice used in the experimental condition involved an easy-to-implement 4-step finger movement procedure that could be easily administered online. On the first session, the clinical psychologist described the technique, showing each of the four BWM-T finger positions with his own hands, so that participants could mirror them and easily learn them. The first position implied being able to touch the extremity of the little finger with the extremity of the thumb; the second position, being able to touch the extremity of the ring finger and the extremity of the thumb; the third position, being able to touch the extremity of the middle finger and the extremity of the thumb; and the fourth position, being able to touch the extremity of the middle finger and ring finger with the extremity of the thumb. Each position had to be kept for at least 3 min; the psychologist would remind participants to change position when the time expired. Moreover, participants were asked to place their computer or smartphone in a quiet, relaxing place of their homes, and to sit in front of the screen on an armchair or a comfortable chair with the legs placed on the ground and the back aligned to the seatback. Then, they were asked to put both hands on the legs or on the armrest of the chair and keep eyes closed (Cozzolino et al., 2020a,b). The same procedure was used for the next sessions.

On each session, all participants joined in the general online meeting at the same time. Participants in the experimental group stayed in the online room with the study clinical psychologist and received the intervention. As the platform used for the online intervention only allow to display up to 49 participants per screen, four research assistants were in charge of monitoring the participants during the intervention (each one monitoring a quarter of participants), in order to verify whether their positions were consistent with the technique, while the psychologist was focused in carrying out the intervention. Controls were asked to enter another online room, where another study researcher shared the videos on stress reduction with participants. The study started on week four of the first quarantine in Italy (week commencing 30th March 2020) and ended on April 24th, before lockdown measures were loosened. Of the 310 participants, 266 (85.8%) completed the questionnaire at both time-points.

### Analysis

First of all, differences between the experimental and the control group were estimated with regard to demographic statistics and the key variables of the study. Than, for descriptive purposes, differences in gender distribution among remainers and dropouts were calculated performing a Chi-square analysis. Univariate analyses of variance on age and all the key variables of the study were computed with study drop-out as the independent variable and the baseline assessments as dependent variables (age, distress, perceived stress, anxiety, global distress, negative and positive affect). In order to evaluate the effects of the BWM-T, a series of two-factor mixed-design analysis of variance (ANOVA) were conducted using time as within-subjects factor (pre- vs. post-intervention) and conditions as between-subjects factor (experimental vs. control). The 2 × 2 mixed-design ANOVA was conducted separately for each variable of the study, using the IBM SPSS Statistics for Windows, version 23 (IBM Corp., Armonk, N.Y., United States).

## Results

### Preliminary Descriptive and Attrition Analyses

No significant differences before the intervention were found between the two groups (experimental and control) neither for demographic characteristics [gender: *X*^2^_(1)_ = 0.47; *p* = 0.49; age: *F*_(1, 309)_ = 0.008; *p* = 0.927], nor for the key variables of the study [DT: *F*_(1, 310)_ = 1.314; *p* = 0.25; PSS: *F*_(1, 303)_ = 0.715; *p* = 0.395; PA: *F*_(1, 300)_ = 0.494; *p* = 0.48; NA: *F*_(1, 300)_ = 0.79; *p* = 0.37; global distress: *F*_(1, 300)_ = 0.077; *p* = 0.78; trait-anxiety: *F*_(1, 296)_ = 1.572; *p* = 0.21; state-anxiety: *F*_(1, 296)_ = 2.971; *p* = 0.09]. No significant difference between remainers and dropouts emerged with respect to gender distribution [*X*^2^_(1)_ = 2.20; *p* = 0.13]. The results of the preliminary analysis indicated that those who remained in the study at final time-point differed on age, trait-anxiety, global distress, and positive affect, compared to those who dropped out. Remainers were older [*F*_(1, 307)_ = 18.65; *p* < 0.001; *M* age = 29.63; *SD* = 9.27; *M* age = 23.36; *SD* = 6.23. respectively], had lower levels of trait-anxiety [*F*_(1, 294)_ = 5.49; *p* < 0.05; *M* = 46.05; *SD* = 10.96; *M* = 50.47; *SD* = 10.05, respectively], lower levels of global distress [*F*_(1, 300)_ = 4.34; *p* < 0.05; *M* = 17.13; *SD* = 6.08; *M* = 19.30; *SD* = 6.37, respectively], and lower levels of positive affect [*F*_(1, 300)_ = 5.07; *p* < 0.05; *M* = 28.77; *SD* = 6.62; *M* = 31.32; *SD* = 6.91, respectively], than those who dropped out. No significant differences between remainers and dropouts emerged with respect to perceived stress (measured with either DT or PSS), state-anxiety, and negative affect.

### Changes in Perceived Stress, Subjective Well-Being, Global Distress and Anxiety

Table 1 illustrates the results of the 2 × 2 mixed-design factorial ANOVA, means and standard deviations for all the constructs under examination in pre- and post-intervention, for both conditions. Findings showed a statistically significant effect of the interaction between time and condition in participants’ distress and perceived stress both measured via DT and with PSS, in the expected direction: participants in the experimental condition had a reduction in perceived stress compared to participants in the control condition. Findings of the 2 × 2 mixed-design factorial ANOVA showed statistically significant effects of the interaction between time and condition in participants’ trait-anxiety, state-anxiety, and global distress, which were also in the expected direction: participants in the experimental condition had a reduction in trait-anxiety, state-anxiety and global distress, compared to participants in the control condition. Finally, findings of the 2 × 2 mixed-design factorial ANOVA also showed statistically significant effects of the interaction between time and condition in participants’ negative affect and positive affect which were in the expected direction: participants in the experimental condition had a reduction in negative affect and an increment in positive affect compared to participants in the control condition.

**TABLE 1:** Means (standard deviations) of the key variables of the study for both conditions (Experimental vs. Control) and time (Pre vs. Post-Intervention).

	Intervention group		Control group		Interaction effect time x group (*F*-value)		
			
	*M*	*SD*	*M*	*SD*	*F*	*df*	η^2^
**Distress**							
Pre	6.87	2.0	6.61	1.98	36.37***	1; 264	.12
Post	4.90	2.25	6.27	2.23			
**Perceived stress**							
Pre	23.27	6.12	22.65	6.49	15.93***	1; 261	.05
Post	19.51	6.40	21.40	6.58			
**S-Anxiety**							
Pre	49.76	12.83	47.23	12.26	22.01***	1; 255	.08
Post	42.97	12.11	47.65	12.80			
**T-Anxiety**							
Pre	47.34	11.31	45.74	10.43	7.47**	1; 256	.03
Post	45.06	10.63	45.55	11.41			
**Global distress**							
Pre	17.50	6.23	17.31	6.08	17.07***	1; 260	.06
Post	14.17	6.63	16.93	6.53			
**Negative affect**							
Pre	30.44	7.13	29.71	6.91	30.59***	1; 260	.10
Post	24.99	7.04	28.30	7.20			
**Positive affect**							
Pre	28.86	6.66	29.41	6.78	7.79**	1; 260	.02
Post	31.72	6.61	30.67	6.96			

**p < 0.01; ***p < 0.001.

## Discussion

In this study, we evaluated an innovative, neuroscientific based mind-body intervention named the brain wave modulation technique (BWM-T). The BWM-T has several advantages over traditional mind-body interventions. For example, it is a low-cost, sustainable, and reproducible procedure that can be performed in minutes; no special premises or specific equipment are required; it applies to both individuals and groups; it takes just one clinical psychologist, which simplifies scheduling; and, once learned, individuals can perform it autonomously at home (Cozzolino et al., 2020a,b). The study’s findings support our hypothesis that participants who attended a 4 weeks BMW-T intervention, administered online, would show a reduction in the perception of stress, state-trait anxiety, global distress, and negative affect, as well as an increase in positive affect, compared with controls, who watched videos that gave suggestions on how to reduce stress. These findings are in line with previous studies, which have suggested the effectiveness of mind-body therapies in reducing stress (Deckro et al., 2002; Hagen and Nayar, 2014; Gallego et al., 2016; Lolla, 2018; Zhang et al., 2018). Our intervention also had clinical relevance because the high mean scores for state anxiety, found in all participants before the intervention, fell into the nonclinical range for adults in the experimental participants after the intervention. Interestingly, the mean scores for trait anxiety also decreased in this group. This is likely due to the fact that the technique we used in our intervention promotes rebalancing of the states of physiological activation and relaxation, which are often impaired in subjects who show high trait anxiety (Murata et al., 2004; Desai et al., 2015; Lee et al., 2015; Das, 2019; Cozzolino et al., 2020a,b). The BWM-T modulates brain waves from fast and intensive to slower and larger waves that are typical of relaxation and deep sleep (Cozzolino and Celia, 2016; Rocco et al., 2018; Celia and Cozzolino, 2021). Moreover, since the effects were obtained through an online administration of the technique, our findings confirm and expand on previous research that investigated the effects of a face-to-face BWM-T intervention (Cozzolino et al., 2020a,b). We acknowledge that the present study has limitations. First, the sample size was limited. Second, the study included only students and the vast majority of them were girls, therefore caution should be made in the interpretation of the results. It would be interesting to replicate this study in a larger sample size, with different target populations and with a sample more balanced in terms of gender distribution. Moreover, we did not take into account history and local history effects since the experiment was carried out during the COVID-19 pandemic in Italy.

## Conclusion

Traditional interventions for stress and anxiety often rely more heavily on relational aspects and are therefore less suited for use remotely in emergency situations such as the pandemic. This is a particularly important advantage of the BWM-T, because managing the long-term psychological impact of COVID-19 pandemic requires effective evidence-based therapies that can also be favorably administered online for those who will continue to have difficulties accessing face-to-face mental health services. Further research should explore the sustainability of the effects of the BWM-T over time and in different categories of individuals across all age ranges, from children to the elderly.

## Data Availability Statement

The raw data supporting the conclusions of this article will be made available by the authors, without undue reservation.

## Ethics Statement

Ethical review and approval was not required for the study on human participants in accordance with the local legislation and institutional requirements. The patients/participants provided their written informed consent to participate in this study.

## Author Contributions

MC and GC made the greatest contribution to the manuscript, designing the research, drafting the work, and contributing to all the steps of the work. LG conducted the statistical analyses. PL revised the manuscript and monitored all the process providing scientific and theoretical contribution. All authors approved the final version of the manuscript and agreed to be accountable for all aspects of the work in ensuring that questions related to the accuracy or integrity of any part of the work are appropriately investigated and resolved.

## Conflict of Interest

The authors declare that the research was conducted in the absence of any commercial or financial relationships that could be construed as a potential conflict of interest.
