# Supplementary material for: Effects of the Brain Wave Modulation Technique Administered Online on Stress, Anxiety, Global Distress, and Affect During the First Wave of the COVID-19 Pandemic: A Randomized Clinical Trial
Source: Front Psychol. 2021 May 19;12:635877. doi: 10.3389/fpsyg.2021.635877 (PMC8170086; doi:10.3389/fpsyg.2021.635877)
Supplement: Supplementary file 1 [file Table_1.DOCX]

**APPENDIX A**

**THE ADMINISTRATION OF THE BRAIN WAVE MODULATION TECHNIQUE**

Find a relaxing place where no causes of disturbance are present (such as noise or vibrating/ringing phones). Sit on an armchair or a comfortable chair with your legs placed on the ground and the back

aligned to the seatback. Put both hands on the legs or on the armrest of the chair and keep eyes closed.

- First phase: Be able to touch the extremity of the little finger with the extremity of the thumb
- Second phase: Be able to touch the extremity of the ring finger and the extremity of the thumb
- Third phase: Be able to touch the extremity of the middle finger and the extremity of the thumb
- Fourth phase: Be able to touch the extremity of the middle finger and ring finger with the extremity of the thumb

All the phases must be done with both left and right hand simultaneously. It is important that the extremities touch each other perfectly (Cozzolino & Celia, 2016).
